# Effects of Qingshen Granules on the Oxidative Stress-NF/kB Signal Pathway in Unilateral Ureteral Obstruction Rats

**DOI:** 10.1155/2018/4761925

**Published:** 2018-02-08

**Authors:** Hua Jin, Yiping Wang, Dong Wang, Lei Zhang

**Affiliations:** Department of Nephrology, The First Affiliated Hospital of Anhui University of Traditional Chinese Medicine, Hefei 230031, China

## Abstract

*Background*. The activation of NF-kappa B (NF/kB) signaling pathway plays an important role in the process of epithelial-mesenchymal transition (EMT) and renal interstitial fibrosis (RIF) in renal tubules. The process of oxidative stress reaction in kidney is via excessive reactive oxygen species (ROS) production to activate NF/kB signaling pathway. Qingshen Granule (QSG) is an effective Chinese formula utilized to treat chronic renal failure. Previous studies confirmed that QSG could inhibit RIF in unilateral ureteral obstruction (UUO) rats. In this study, we used UUO rats to investigate the effects of QSG on oxidative stress and the activation of NF/kB signaling. Seventy male Sprague-Dawley (SD) rats were randomly divided into a sham group, UUO model group, Qingshen Granules (QSG) high-dose, medium-dose, and low-dose groups, PDTC group, and candesartan group (10 rats in each group). Our study demonstrated that oxidative stress-NF/kB signal pathway contributed to the formation of UUO renal interstitial fibrosis. QSG may protect against RIF by inhibiting the oxidative stress-NF/kB signal pathway, reducing inflammation, and improving renal tubular EMT.

## 1. Introduction

Chronic kidney disease (CKD) often progresses slowly which leads to an extensive RIF in renal tissue and finally into end-stage renal disease (ESRD) [1–3]. The main pathological features of renal fibrosis are glomerular sclerosis, renal interstitial fibrosis, and renal vascular sclerosis [4]. The degree of tubulointerstitial lesions has a decisive influence on the survival rate of the kidney and is more important in the prognosis of renal than glomerular disease. A large number of experimental and clinical studies have found that EMT is one of the key processes that lead to RIF [5–8].

Previous studies have shown that the activation of the NF-kappa B (NF/kB) signaling pathway plays an important role in the process of EMT and RIF in renal tubules. The NF/kB signaling pathway is the main control factor in regulating immune and inflammatory responses. The activation of the NF/kB signaling pathway by an oxidative stress reaction in the kidney via excessive ROS production leads to renal inflammatory immune response, tubular EMT, extracellular matrix (ECM) accumulation, and, finally, RIF. Therefore, additional research on the mechanism of oxidative stress mediated activation of the NF/kB signaling pathway and the mechanism of renal tubular EMT should be conducted because it may contribute to improving the treatment of RIF.

In a clinical study [9], Wang et al. show that QSG can be effective in the treatment of chronic kidney disease. In our previous study, we confirmed that QSG can inhibit RIF in model-unilateral ureteral obstruction (UUO) rats [10]. Whether this effect is achieved by inhibiting the renal oxidative stress signal channel to delay RIF is still unknown. Therefore, in order to offer a theoretical basis for the anti-RIF effect of QSG, in this study, we use rats with RIF induced by unilateral ureteral obstruction (UUO) to investigate the effects of QSG on oxidative stress and on the activation of NF/kB signaling.

## 2. Materials and Methods

### 2.1. Animals

Pathogen-free male Sprague-Dawley rats weighing 180–200 g were purchased from Beijing Vital River Laboratory Animal Technology Co. Ltd. [license number: SCXK (Jing) 2012-0001] and reared in the Key Laboratory of Xin'an Medicine (Ministry of Education). The animal experiments were reviewed and approved by the Animal Care and Use Committee of Anhui University of Chinese Medicine. All animals were maintained with a standard laboratory diet under controlled indoor temperature (23 ± 1°C) and humidity (65–70%) conditions. The study was performed strictly according to the guidelines developed by the National Research Council's* “Guide for the Care and Use of Laboratory Animals”* and was approved by the Experimental Animal Care and Ethics committees of Anhui University of Chinese Medicine (He Fei, Anhui, China). Every effort was made to minimize suffering.

### 2.2. Modeling and Grouping

Animals were anesthetized with 10% hydrate of chlorine aldehyde (0.3 m1/100 g body weight), and their left ureter was exposed and separated through a flank incision. In the rats undergoing UUO, the left ureter was ligated with 4-0 silk at two points and then severed between the ligatures, whereas in the sham-operated rats, the left ureter was left undisturbed. The rats were randomly divided into the following seven groups by random number table method: (1) sham group (healthy control rats, *n* = 10); (2) model group (UUO rats, *n* = 10); (3) QSG high-dose group (UUO rats, *n* = 10); (4) QSG medium-dose group (UUO rats, *n* = 10); (5) QSG low-dose group (UUO rats, *n* = 10); (6) PDTC group (UUO rats, *n* = 10); (7) candesartan group (UUO rats, *n* = 10).

### 2.3. Drug Administration

Drug administration began on the 7th day after the surgery. Qingshen Granules (QSG) bags (10 g/bag) were provided by the First Affiliated Hospital of Anhui Medical Center (Hefei, China, batch number: 20141023). Candesartan tablets (4 mg/tablet) were obtained from Chongqing Shenghuaxi Pharmaceutical Limited by Share Ltd. (Chongqing, China, batch number: H20030771). PDTC was obtained from Sigma company (USA, batch number: AB141406; 5 g/bottle). Following past studies [11], the rats in both the sham and model groups were given 0.9% physiological saline (1 mL/100 g per day). The rats in the QSG group were given 4.0 g/kg·d, 8.0 g/kg·d, and 16.0 g/kg·d in low-dose, medium-dose, and high-dose groups, respectively. The rats in the candesartan group were given 1.0 mg/kg bodyweight candesartan per day. The rats in the PDTC group received intraperitoneal injections of 40 mg/kg bodyweight PDTC per day, dissolved in warm water. The total intervention time was four weeks.

### 2.4. Sample Collection

After euthanizing the rats, blood was taken from the abdominal aorta and then placed in the common vacuum vessel. Fifteen minutes of centrifugation with a low speed centrifuge (3000 r/min) followed. The serum was collected in the Eppendorf tube, which was kept in the −80°C refrigerator. The right kidneys were removed, some fresh tissue samples were taken from them, weighed, and placed in freezing tube (per 0.1 g tissue add 1 ml phosphate buffer, PH 7.4). The remaining kidney tissues were fixed in 10% neutral formaldehyde solution.

### 2.5. Instruments

The instruments used are as follows: microplate reader (manufacturer: Rayto, model: RT-6000, China); microplate washer (manufacturer: Rayto, model: RT-3100, China); electric heating constant temperature (manufacturer: San Fa Scientific Instrument Co., Ltd., model: DNP-9052BS- III, China); centrifuge (manufacturer: Sigma company, model: 2-16PK, Germany); −80°C refrigerator (manufacturer: Thermo Fisher Scientific, model: Thermo-700, USA); pathological section machine (manufacturer: Leica, model: LEICA-213, Germany); paraffin embedding station (manufacturer: Leica, model: LEICA-115, Germany); microscope (manufacturer: Xinfeida Optical Instrument Company, model: Nikon80, China).

### 2.6. ELISA Analysis

Serum concentrations of creatinine were determined by using a creatinine enzyme-linked immunoassay kit (manufacturer: Nanjing Jiancheng Bioengineering Institute, batch number: 20151102, China). Renal tissue concentrations of reactive oxygen species (ROS), superoxide dismutase (SOD), and malondialdehyde (MDA) were determined using commercially available assay kits (manufacturer: Shanghai Yuanye Bio-Technology Co., Ltd., batch number: 20151018A, 20151026A, 20151102A, China), according to the manufacturer's instructions.

### 2.7. Western Blot Analysis

Operations were carried out strictly according to kit instructions. The entire process of producing a Western blot includes sample preparation, gel electrophoresis, transfer from gel to membrane, and immunostain of the blot. The primary antibodies were added according to the manual and with the following dilutions: MCP-1 (batch number: 131120w, manufacturer: Beijing Bioss Biotechnology Co., Ltd., China) 1 : 250, ICAM-1 (batch number: 00025688, manufacturer: Proteintech Group, USA) 1 : 250, NF-*κ*Bp65 (batch number: GR194772-1, manufacturer: Abcam, England) 1 : 2000, p-I*κ*B*α* (batch number: CJ44121, manufacturer: Bioworld Technology, Inc., USA) 1 : 500, IKK*α* (batch number: AA54131, manufacturer: Bioworld Technology, Inc., USA) 1 : 500, and Sheep-Horseradish Peroxidase Anti-Mouse IgG (batch number: 109525, manufacturer: Beijing Zhongshan Biological Technology Co. Ltd., China) 1 : 1000. An automated gel imaging system (manufacturer: Beijing Kechuang Ruixin Biological Technology Co. Ltd., model: K8360, China) was used for the analysis strips.

### 2.8. Immunohistochemical Analysis

The slides were deparaffinized in xylene and hydrated with graded alcohol (100–80%). After washing one time with phosphate-buffered saline (PBS) for 30 min at room temperature, the slides were warmed in microwave with citrate buffer. After cooling, the slides were washed three times in distilled water (DW) for five minutes each, following addition of primary antibodies 3% bovine serum albumin (BSA) in PBS and incubated overnight at 4°C. After washing with DW, the slides were incubated with secondary antibodies for 1 h. Subsequently, the sections were washed in DW and incubated for 30 sec to 1 min at room temperature with 3,3′-diaminobenzidine (DAB) as selenium organic reagent. Lastly, counterstaining was performed with hematoxylin solution. All the slides were washed with xylene and mounted using Permount mounting medium. The slides were then examined under a light microscope. Image Pro Plus multimedia color image analysis software 6.0 (USA) was used to analyze the average optical density of positive expression.

### 2.9. Histopathology

The kidney tissues were fixed in 10% neutral formaldehyde solution and embedded in paraffin for histological evaluation. Paraffin-embedded renal samples were sectioned, prepared at a thickness of 2 um, and placed on glass slides. The slides were stained with hematoxylin and eosin (H&E) and Masson's trichrome for histopathological analysis. The operation of staining the samples was according to the instructions. To assess renal tubulointerstitial injury and collagen deposition, the semiquantitative analysis of HE and Masson's stain was evaluated as described previously [12].

### 2.10. Statistical Analysis

Continuous variables are expressed as mean ± standard deviation. All samples were tested to ascertain if they followed a normal distribution. The sample data have a normal distribution. Data comparison among groups was performed using ANOVA and homogeneity of variance tests. Comparisons between groups were carried out using the independent samples *t*-test. SPSS version 17.0 (SPSS Inc., Chicago, Illinois, USA) was used for data analyses. *P* < 0.05 was considered significant.

## 3. Results

### 3.1. Comparisons of Serum Creatinine Level with ROS, SOD, and MDA Levels in Renal Tissue

**Figure 1:**
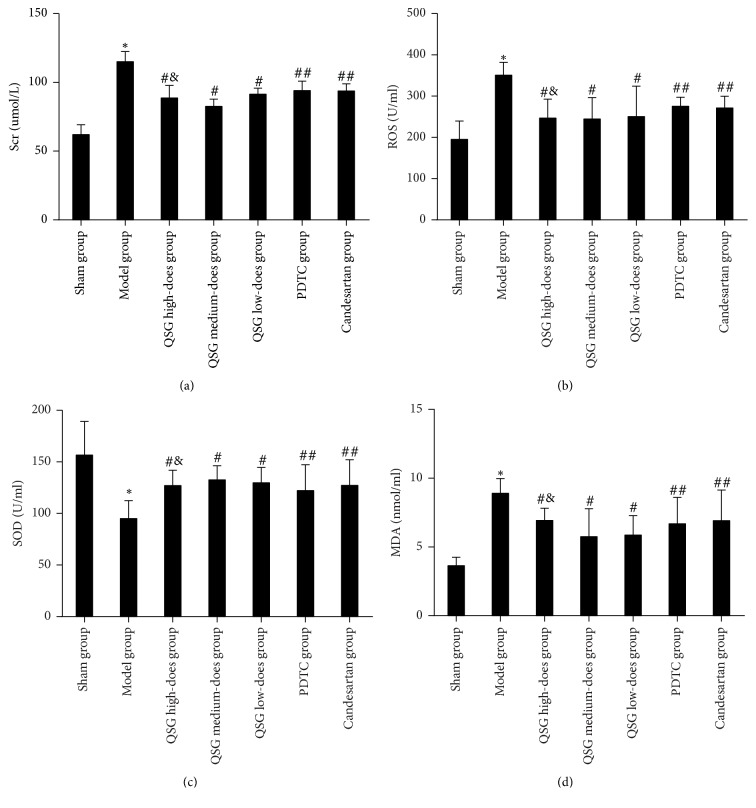
*Comparison of serum creatinine levels with levels of ROS, SOD, and MDA in each group*.* Notes*. ^*∗*^ *P* < 0.01, compared with the sham group; ^#^ *P* < 0.01, ^##^ *P* < 0.05, compared with the model group; ^&^ *P* < 0.05, compared with the PDTC and candesartan groups.

Figure 1(a) shows that, compared with the sham group, the serum creatinine level in the model group was higher (*P* < 0.01) but lower in the QSG high, medium, and low-dose groups (*P* < 0.01). Moreover, the serum creatinine in PDTC group and candesartan groups was lower than that of model group (*P* < 0.05), and the serum creatinine level in the QSG medium-dose group was the lowest (*P* < 0.05). Figures 1(b) and 1(d) show that the concentrations of MDA and ROS in the kidney tissue in the model group were higher (*P* < 0.01) than those in the sham group. In the QSG, PDTC, and candesartan groups, however, the levels of MDA and ROS were lower than in the model group (*P* < 0.01); the lowest levels were found in the QSG medium-dose group (*P* < 0.05).  Figure 1(c) illustrates that the concentration of SOD in the kidney tissue in the model group was lower (*P* < 0.01) than in the sham group. Moreover, it shows that the SOD concentration in the QSG, PDTC, and candesartan groups was higher than that in the model group (*P* < 0.01, *P* < 0.05) and that the QSG medium-dose group has the highest SOD concentration (*P* < 0.05).

### 3.2. Expression of ICAM-1, MCP-1, NF-KBp65, p-IKB*α*, and IKK*α*in in the Renal Tissue

**Figure 2:**
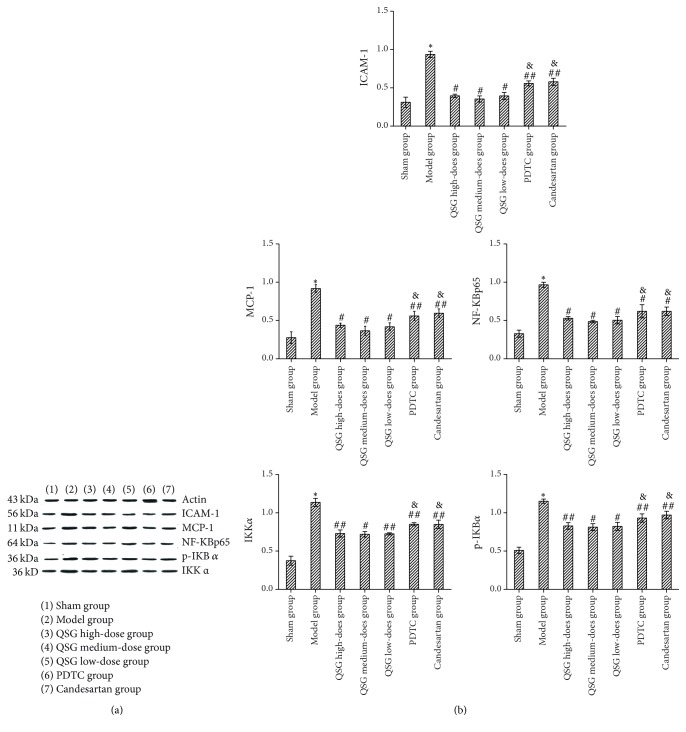
*Expression of ICAM-1, MCP-1, NF-KBp65, p-IKBα, and IKKα in each group*.* Notes*. (a) Original strip chart of Western blot results; (b) relative expression quantity of each protein; ^*∗*^ *P* < 0.01, compared with the sham group; ^#^ *P* < 0.01, ^##^ *P* < 0.05, compared with the model group; ^&^ *P* < 0.05, compared with the QSG medium-dose group.

Figure 2(b) shows he original strip chart of the Western blot results. Figure 2(b) shows the relative protein expression. The relative expression of ICAM-1, MCP-1, NF-KBp65, p-IKB*α*, and IKK*α*in in the model group was higher than that in the sham group (*P* < 0.01); in the QSG, PDTC, and candesartan groups the relative expression of those proteins was lower than in the model group (*P* < 0.05). The relative expression of those proteins was the lowest in the QSG medium-dose group (*P* < 0.05).

### 3.3. Comparisons of Immunohistochemistry

**Figure 3:**
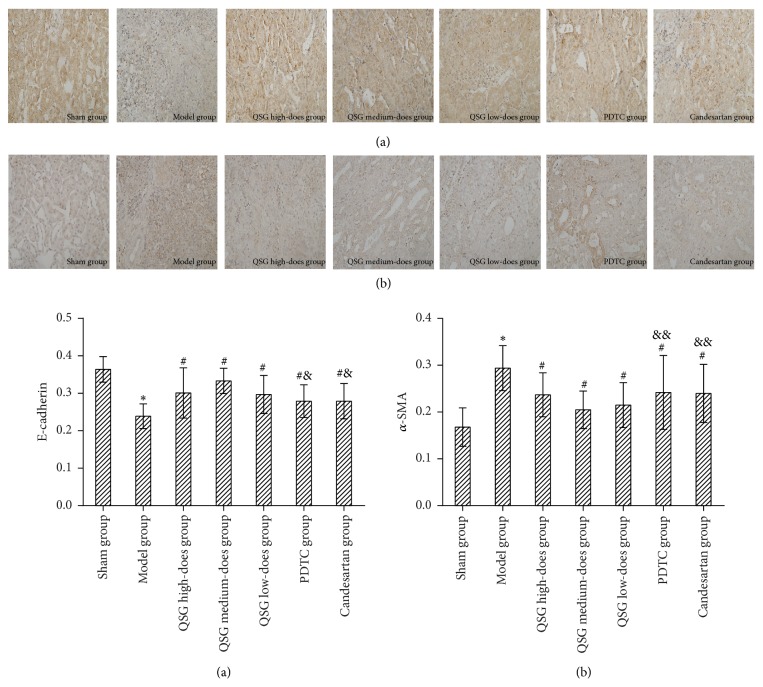
*Comparison of immunohistochemistry and average optical density of E-cadherin and α-SMA protein in each group*.* Notes*. (a) Immunohistochemistry of E-cadherin protein; (b) immunohistochemistry of *α*-SMA protein. ^*∗*^ *P* < 0.01, compared with the sham group; ^#^ *P* < 0.01, compared with the model group; ^&^ *P* < 0.01, ^&&^ *P* < 0.05, compared with the QSG medium-dose group. (×400).

Figure 3 shows the immunohistochemical results of E-cadherin and *α*-SMA protein and its average optical density. Compared with the sham group, the expression of E-cadherin protein in the model group was significantly lower (*P* < 0.01), while the expression of *α*-SMA protein was significantly higher (*P* < 0.01). The expression of E-cadherin protein in the QSG, PDTC, and candesartan groups was higher than in the model group (*P* < 0.05); the QSG medium-dose QSG group had the highest level (*P* < 0.05). The expression of *α*-SMA protein in the QSG, PDTC, and candesartan groups was lower than in model group (*P* < 0.05), and QSG medium-dose group had the lowest level (*P* < 0.05).

### 3.4. Comparison of HE with Masson's Stain

**Figure 4:**
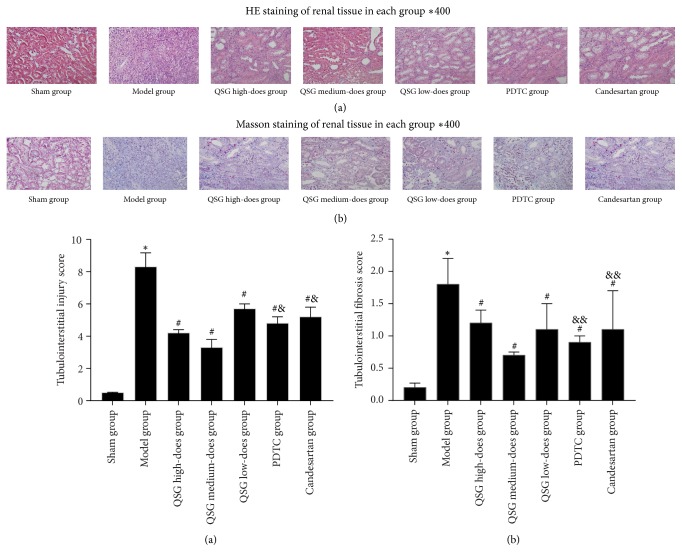
*HE and Masson's stain in each group* ((a) HE, (b) Masson ×400).* Notes*. ^*∗*^ *P* < 0.01, compared with the sham group; ^#^ *P* < 0.01, compared with the model group; ^&^ *P* < 0.01, ^&&^ *P* < 0.05, compared with the QSG medium-dose group.

Figure 4 shows that the size and shape of the renal tubules in the sham group were normal under light microscope. There was no obvious pathological change and less inflammatory cell infiltration in the renal interstitium. In the model group, the tubular structure was destroyed; and renal interstitial edema, renal tubule and collecting duct dilatation, vesicular lumen collapse, and renal tubular epithelial cell necrosis or shedding could be seen. A large number of macrophages and lymphocytes infiltrated in the renal interstitium. Fibroblastic and collagen hyperplasia was found in the renal interstitium. Some renal tubules disappeared and interstitial fibrosis appeared. Blue collagen fibers were clearly visible in the renal interstitium. In all QSG groups, a small number of macrophages and lymphocytes infiltrated into the renal interstitium. The expansion of renal tubules and renal capsule was slight. Compared with the sham group, the tubulointerstitial damage and interstitial fibrosis were significantly higher in the model group (*P* < 0.01); in the QSG, PDTC, and candesartan groups the tubulointerstitial damage and interstitial fibrosis were lower than in the model group (*P* < 0.05). The QSG medium-dose group had suffered the least amount of tubulointerstitial damage and interstitial fibrosis (*P* < 0.05).

## 4. Discussion

RIF is a common development in CKD that can lead to uremia and be life-threatening. Finding effective complementary and alternative medicine for prevention and treatment of RIF is crucial, because current medical approaches do not reliably slow down or reverse RIF. In recent years, traditional Chinese medicine has been widely used in combating RIF [13]. QSG consists of* Herba Hedyotidis Diffusae, Rhizoma Coptidis, Herba Artemisiae Scopariae, Radix et Rhizoma Rhei, Semen Coicis, Rhizoma Atractylodis Macrocephalae, Semen Lablab Album, Poria, Radix Salviae Miltiorrhizae, Herba Leonuri, Rhizoma Alismatis, Polyporus, Herba Plantaginis,* and* Fructus Amomi Rotundus.* It clears away heat, eliminates dampness, and removes blood stasis and turbidity. Clinical studies confirmed that it could not only delay the decline of renal function, but also reduce the level of serum NF-*κ*B p65 and p-I*κ*B*α* in patients with chronic renal failure [14]. Renal fibrosis is the pathological basis of chronic renal failure. EMT has been considered to be one of the key processes in the occurrence and development of RIF. About 30% to 50% of renal interstitial fibroblasts were a product of renal tubular EMT [15]. The UUO model is the classic model of renal fibrosis [16]. About 36% of interstitial fibroblasts in the kidneys of UUO rats stems from renal tubular epithelial cells [17]. *α*-SMA protein, which is mainly used for evaluating the transconformation degree of renal tubular epithelial cells to myofibroblasts, is considered to be a marker protein for the transformation of cells into myofibroblasts [18]. In normal kidney tissues, only the vascular smooth muscle cells express *α*-SMA protein; increased expression of it indicates a transformation to the proliferative phenotype. Expression of this protein by renal tubular epithelial cells indicates that renal tubular epithelial cells have the characteristics of muscle fibroblasts and that a large number of extracellular matrixes, which may participate in the process of RIF, will be secreted [19]. The expression of *α*-SMA protein in the renal tissue of UUO rats was significantly decreased after the intervention with QSG in this study. At the same time, the immunohistochemical results showed that the expression of E-cadherin protein was increased. E-cadherin is a transmembrane glycoprotein found in the cell junction areas. It is a calcium-dependent cell adhesion molecule, with a relative molecular mass of 120000 which contributing to the adhesion between epithelial cells [20]. E-cadherin is expressed in all epithelial cells and is an important adhesion molecule which maintains the polarity and tight junction of renal tubular epithelial cells. If the expression of E-cadherin and tight junction proteins in renal tubular epithelial cells is inhibited, the tight junctions between cells are destroyed and epithelial cells lose their adhesive property and are gradually shed from the basement membrane. In addition, the renal tubular epithelial cells express *α*-SMA protein and other types of mesenchymal proteins. The recombinant proteins are recombined. The tubular basement membrane (TBM) is broken. The migration and invasion of the cells were enhanced. Finally, there is an early change of EMT [21]. After EMT the cells no longer express E-cadherin and tight junction protein but express *α*-SMA protein. Therefore, E-cadherin, *α*-SMA, and tight junction proteins are the markers of EMT and RIF in renal tubules [22, 23]. The levels of E-cadherin and *α*-SMA protein in each group strongly suggest that RIF is effectively inhibited by QSG.

Recent studies have shown that oxidative stress is one of the important causes of kidney damage, especially of tubulointerstitial fibrosis [24]. The increase of oxidative stress reaction products (mainly ROS) may play a very important role in the tubulointerstitial lesions in UUO rats; the inhibition of oxidative stress can alleviate the RIF process [25]. ROS not only can directly play a role in cytotoxicity, but also can be used as a signal molecule to regulate the expression of some related genes. NF/kB is a transcription factor which is closely related to oxidative stress [26]. Superoxide dismutase (SOD) is an important enzyme that defends the injury of superoxide anion in the internal and external environment and that has a super antioxidant effect. The activity of SOD reflects the ability of the body to scavenge oxygen free radicals. Malondialdehyde (MDA) is a common lipid peroxidation product and reflects the level of ROS produced by lipid oxidation, which, in turn, indirectly reflects the degree of oxidative damage in tissues and cells. Therefore, SOD and MDA are commonly used to evaluate the level of oxidative stress in cells [27]. It could be shown that the internal oxidation became unbalanced as the level of blood Scr rose. The levels of ROS and MDA were significantly increased, while the level of SOD was decreased. After the intervention with QSG, this imbalances were corrected.

When oxidative stress occurs, ROS enhances the activation of NF-kB pathways. The activation of NF-kB may induce apoptosis and the expression of inflammatory factors such as intercellular adhesion molecules (ICAM-1) and monocyte chemotactic factor (MCP-1), thereby promoting monocyte adhesion and release of inflammatory factors. The production and release of these inflammatory factors, in turn, further activate NF-kB pathways. The initial inflammatory signal is thus amplified, the time is prolonged, and the local inflammatory response and oxidative stress are further aggravated. More importantly, the inflammatory response is out of control. The above-mentioned mechanism triggers the formation of renal tubular EMT, form myofibroblasts, and begins to secrete and synthesize a series of collagen-fibrin types, resulting in the increase and degradation of the ECM and, ultimately, in RIF [28]. In the resting state of cells, NF-kB forms a dimer with its inhibitory protein I*Κ*B*α* and exists in the cytoplasm in an inactive form. When cells are activated by NF-kB inducers, the two dimers are deactivated. NF-kB is dissociated from I*Κ*B*α* which is phosphorylated (p-IKB*α*) and then degraded. While NF-kB is activated, P56 enters the cell and combines with DNA to activate the downstream target gene. We found that the expression of NF-KBp65, p-IKB*α*, and IKK*α*protein were increased in the tissues of UUO rats. QSG could reduce the levels of those proteins in UUO rat.

Pyrrolidine dithiocarbamate (PDTC) is a specific inhibitor of NF/kB pathway. Our results were in agreement with previous reports [29]. The intervention with PDC led to the inhibition of the inflammatory response and the decrease in the expression levels of MCP-1 and ICAM-1. ACEI and ARB are classic drugs for the suppression of renal fibrosis [30]. Studies on candesartan, one of the ARB drugs, proved it to inhibit not only renal fibrosis but also the inflammatory signaling pathway [31, 32]. Our results confirmed that QSG, especially in the medium-dose group, was more effective in inhibiting the oxidative stress-NF/kB signal pathway and reducing inflammation than candesartan and PDTC.

## 5. Conclusions

This study confirmed that the oxidative stress-NF/kB signal pathway is involved in the formation process of the UUO RIF. The results of this study confirm the results of our clinical study [29] and indicate that the anti-RIF effect of QSG may be based on the inhibition of oxidative stress-NF/kB signal pathway which reduces inflammation and renal tubular EMT and, ultimately, delays the progression of RIF.

## Abbreviations

BSA: Bovine serum albumin
CKD: Chronic kidney disease
DAB: Diaminobenzidine
ECM: Extracellular matrix
EMT: Epithelial-mesenchymal transition
ESRD: End-stage renal disease
MDA: Malondialdehyde
PBS: Phosphate-buffered saline
PDTC: Pyrrolidine dithiocarbamate
QSG: Qingshen Granules
RIF: Renal interstitial fibrosis
ROS: Reactive oxygen species
SD: Sprague-Dawley
SOD: Superoxide dismutase
UUO: Unilateral ureteral obstruction.
